# Assessing executive functions and metacognition: translational potential of the Metacognitive Wisconsin Card Sorting Test for developmental neuropsychology

**DOI:** 10.3389/fnbeh.2025.1655310

**Published:** 2025-09-04

**Authors:** Giovanni Granato, Giulia Manzi, Jordy Di Giulio, Concetto Puzzo, Andrea Mattera, Walter Adriani, Gianluca Baldassarre, Micaela Capobianco

**Affiliations:** ^1^Laboratory of Embodied Natural and Artificial Intelligence, Institute of Cognitive Sciences and Technologies, National Research Council of Italy, Rome, Italy; ^2^Center for Behavioural Sciences and Mental Health, National Institute of Health, Rome, Italy; ^3^Department of Economic, Psychological, and Communication Sciences, Niccolò Cusano University, Rome, Italy

**Keywords:** metacognition, executive functions, neuropsychological assessment, Metacognitive Wisconsin Card Sorting Test, neuro-developmental disorders

## Abstract

The development of metacognition and executive functions supports adaptive and goal-oriented behavior in adulthood. Therefore, effective screening of these skills is essential for implementing early interventions in educational and clinical settings. While neuropsychological tests usually focus on a single skill and require clinicians to use lengthy batteries, the Metacognitive Wisconsin Card Sorting Test (Meta-WCST) assesses metacognition, executive functions, and their interaction. However, this test has not yet been scientifically validated for children with either typical or atypical development. This gap highlights both a methodological shortcoming and a missed opportunity for developmental neuropsychology. In this review, we provide a comprehensive analysis of studies involving the Meta-WCST, aiming to evaluate its translational potential for developmental applications. Despite several methodological limitations in the current literature, our evaluation indicates that the Meta-WCST can be adapted to developmental contexts through targeted improvements to theoretical and computational frameworks, data analysis methods, and protocol procedures. These considerations have meaningful implications for multiple areas of developmental neuropsychology, including scientific research, educational practices, and clinical assessments.

## 1 Introduction

Metacognition and executive functions facilitate adaptable and goal-oriented behavior throughout the lifespan. In detail, metacognition (MC) is conceptualized as the monitoring (metacognitive monitoring; MC-M) and control (metacognitive control; MC-C) of one's neurocognitive processes (Nelson and Narens, 1994; Flavell, 1979). The maturation of these components is non-uniform along growth and shows temporal discrepancies (Roebers, 2017). On the other hand, executive functions (EF) are key processes that support goal-directed decision making, strategy adaptation, and problem solving (Diamond, 2013). Similar to MC, the development of EF subcomponents (inhibition, working memory, cognitive flexibility) shows a non-uniform maturation (Best and Miller, 2010; Diamond, 2013). Notably, recent reviews (Roebers, 2017; Marulis et al., 2020; Fleming, 2024) highlight that MC and EF strongly interact both throughout development and during adulthood. Furthermore, both MC and EF are related to academic achievement (Roebers, 2017), career success (Chan et al., 2021; Jain et al., 2023), and wellbeing (Luerssen and Ayduk, 2017; Varshney and Barbey, 2021). Therefore, the literature strongly emphasizes the importance of developing early assessments and interventions that consider both educational and clinical contexts. Unfortunately, the majority of psychological and neuropsychological tests target EF (e.g., the Stroop task; MacLeod, 2005) or metacognition (e.g., perceptual decision-making task; Rahnev, 2021), thus failing to provide a satisfactory measure of both constructs and their interaction. This limitation forces researchers and clinicians to exploit multiple test batteries, thus making developmental experiments and assessments time-consuming and hard to interpret (Howieson, 2019). Notably, the Metacognitive Wisconsin Card Sorting Test (meta-WCST; Koren et al., 2004) introduces a modified version of the most widely used test of cognitive flexibility (WCST; Berg, 1948; Heaton et al., 1993), thereby examining the relationship between executive functioning and MC sub-components (MC-M and MC-C). Unlike the basic WCST, this version has only been validated in adults (Koren et al., 2004, 2005, 2006; Arbel et al., 2013; Marucci et al., 2019; Tercero et al., 2021; Badal et al., 2023b,a; Gorora et al., 2024; Jones et al., 2021; Quiles et al., 2014; Bampa et al., 2024; Fan et al., 2025) and teenagers (Koren et al., 2017), with no validation in children with typical and atypical development (e.g., ADHD, SLD, autism). This methodological gap significantly limits the use of the test in developmental research, educational programs, and clinical evaluations. We address these issues by conducting a comprehensive review of studies involving the meta-WCST administration and/or its data analysis. This review has resulted in an evaluation of the task's adaptability to developmental contexts, leading to the proposal of specific directions for this adaptation. Overall, our evaluations have implications for several fields of developmental neuropsychology, thus offering insights for the development of an assessment tool for educational and clinical contexts. Furthermore, they stimulate the improvement of task-related analysis, enhancing its usability for research applications with populations of all ages.

## 2 The task

**Figure 1:**
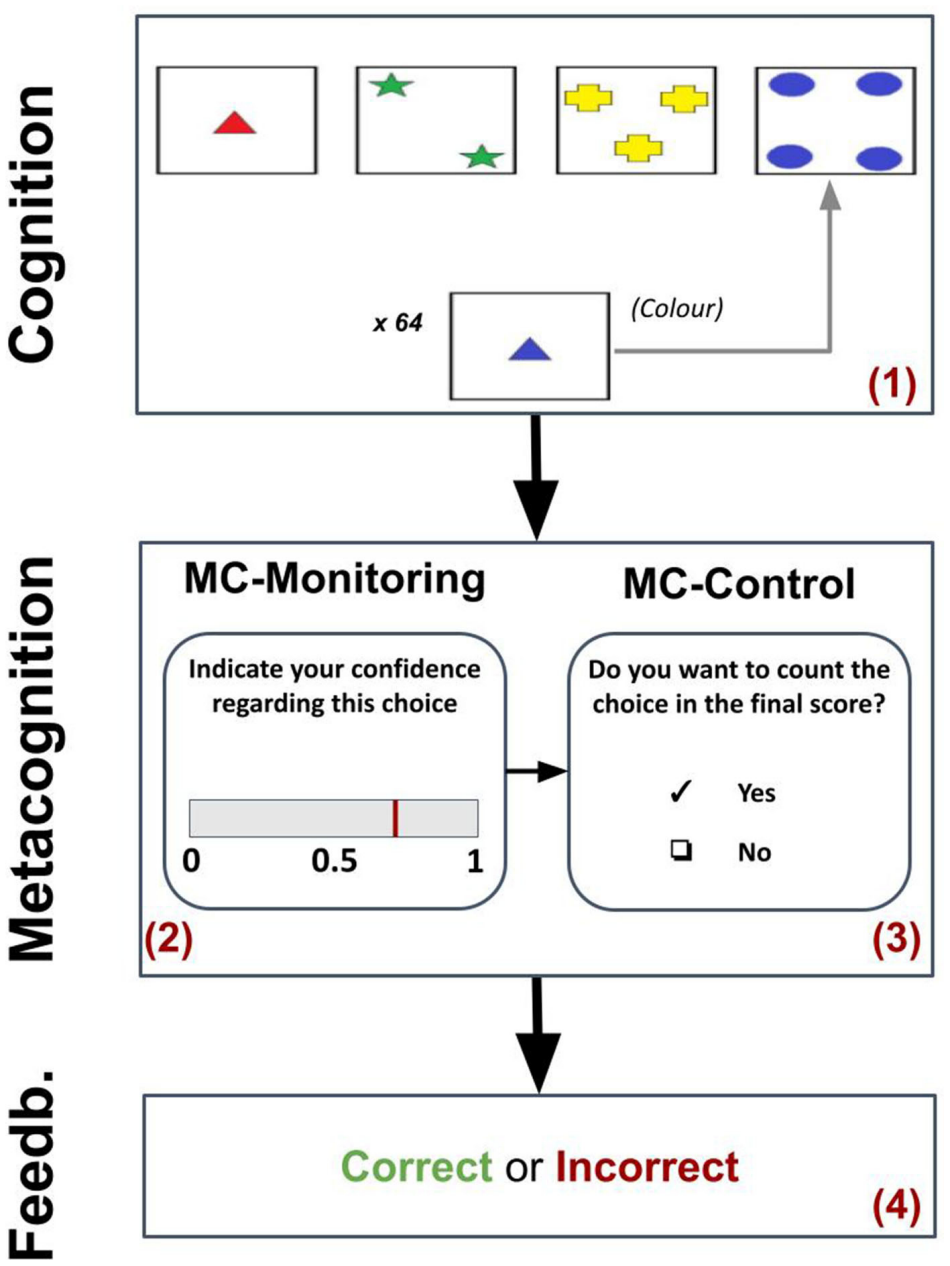
Task protocol. The diagram shows the four phases of a single trial (numbered in red), highlighting the basic neuropsychological processes (cognition, metacognition, and feedback processing) and the sub-phases (MC-monitoring and MC-control).

The Meta-WCST (Koren et al., 2004, 2006) is an alternative version of the WCST (the gold-standard neuropsychological test of executive functions; Berg, 1948; Heaton et al., 1993). The basic version requires participants to sort 128 cards with items showing a combination of three characteristics/sorting rules (color, shape, number). After each sort, the operator returns feedback (“Yes”/“No”) without informing the participant of the correct rule. Therefore, participants infer the correct rule based on the feedback. Noteworthy, the sorting rule changes without warning after 10 correct sorts, so the participant must adjust their sorting based on the changed feedback. The Meta-WCST introduces the assessment of metacognitive functions (MC-M and MC-C; Flavell, 1979). Unlike the WCST, participants are asked to rate their confidence level in their sorting choice and decide whether that response should be counted or discarded for the final score. Therefore, a single trial consists of (1) a sorting phase, (2) a metacognitive monitoring phase—confidence assignment, (3) a metacognitive control phase—double choice, and (4) a feedback phase (Figure 1). Participants are presented with only 64 cards due to the additional metacognitive task phases.

### 2.1 Experimental indices

The Meta-WCST supports the collection of behavioral indices developed within the WCST (we define them “cognitive indices”) and new behavioral indices reflecting metacognitive processes (we define them “metacognitive indices”).

Here, we summarize a set of key cognitive indices taken from official documentation (Heaton et al., 1993). Correct Responses (CR; correct matches), indicating overall performance. Total Errors (TE; incorrect matches), indicating overall performance. Completed Categories (CC; set of 10 sequential CR), reflecting overall performance and understanding of the task structure. Perseverative Errors (PE; errors based on an incorrect and already completed rule), indicating rigidity. Non-Perseverative Errors (NPE; all errors that are not PE); indicating distraction and reasoning failures. Failures-to-Maintain Set (FMS; errors occurred after five sequential CR), reflecting sustained attentional failures. Learning-to-learn index (LTL): the average of successive differences between error rates for each completed category, reflecting changes in conceptual efficiency and possibly a metacognitive process. Metacognitive indices integrate confidence assignments and metacognitive choices into their computation. Quantity Score (QS; CR / trials), reflecting global performance. Because of its computation, it can be defined as a cognitive index. Accuracy Score (AS; counted CR / counted trials), reflecting MC-M efficiency. Free-choice Improvement (FIM; AS - QS), reflecting the impact of metacognitive measures on cognitive ones. Global monitoring (GM; CR - counted trials), indicating the reliability of MC-M processes. Monetary Gain (MG; counted CR - counted TE), reflecting the interaction between response correctness and MC-C decisions. Monitoring Resolution (MR; correlation between trial-by-trial confidence and response correctness), reflects how well confidence predicts response accuracy. Control Sensitivity (CS; the correlation between trial-by-trial confidence and MC-C decision), reflecting the relationship between MC-M and MC-C processes.

## 3 Theoretical and experimental studies

**Figure 2:**
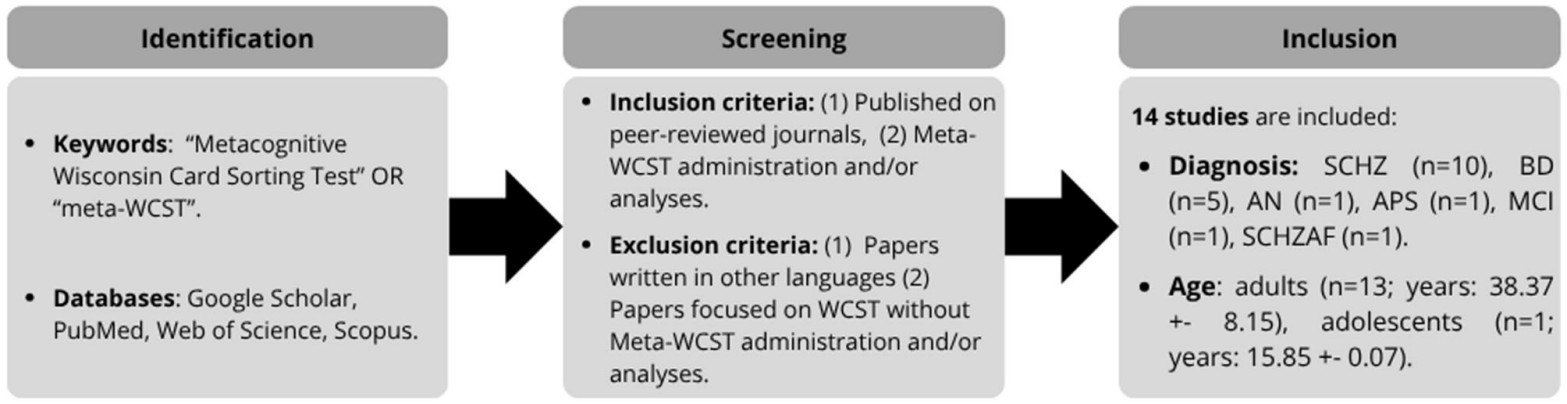
Review process. The diagram shows the three phases of our review process and their criteria/output.

This review focuses on studies involving the administration and/or data analysis of the meta-WCST and was supported by a structured process (Figure 2). Therefore, we performed parallel searches on several databases (Google Scholar, PubMed, Web of Science, Scopus) using the keywords “Meta-WCST” and “Metacognitive Wisconsin Card Sorting Test”. We selected these keywords because our review focused only on the metacognitive version of the WCST, regardless of the population to which it was administered. The search was limited to articles published between January 2004 (the first introduction of the meta-WCST) and May 2025. The eligibility criteria included English-language publications with the following features: (1) being published in peer-reviewed journals, (2) the presence of meta-WCST administration/analyses. The online search was exhaustive, and 14 studies met our inclusion criteria (Table 1). Among them, although several studies included different clinical groups, the majority involved participants with schizophrenia (SCHZ; *n* = 10) and bipolar disorder (BD; *n* = 5). Other clinical conditions were investigated, each in only one study, such as anorexia nervosa (AN), risk of psychosis (APS), mild cognitive impairment (MCI), and schizoaffective disorder (SCHZAF). Overall, healthy controls were included in three studies, and one study involved only healthy individuals. Finally, 13 studies focused on adult populations (38.37 ± 8.15 years), one study on adolescents (15.85 ± 0.07 years), and none on children.

**Table 1:** Meta-WCST studies.

**Reference**	**Focus**	**Group**	**Indices**	**Findings**	**Limitations**
Koren et al. (2004)	Insight, Metacognition	SCHZ	QS, CC, PE, PR, TFC, AS, FIM, GM, MG, MR, CS	constructs correlation	No HC, Sample features
Koren et al. (2005)	Consent comp., Metacognition	SCHZ	QS, CC, PE, PR, TFC, AS, FIM, GM, MG, MR, CS	constructs correlation	No HC, Sample features
Koren et al. (2006)	Insight, Consent comp., Metacognition	SCHZ	QS, CC, PE, PR, TFC, AS, FIM, GM, MG, MR, CS	constructs correlation	No HC, Sample features
Arbel et al. (2013)	Insight, Metacognition	AN, HC	QS, CC, PR, AS, FIM, GM, MG, MR, CS	constructs correlation	Sample features, few cognitive indices
Quiles et al. (2014)	Effect of MC measures on cognition	HC	CC, TE	No effect	Few cognitive indices
Koren et al. (2017)	Self-disturbance, Metacognition	HC, APS	CR, AS, MR, CS	Positive correlation, No APS moderation	No HC, Sample features, No cognitive indices
Marucci et al. (2019)	OME, SME	SCHZ	PE, AS, FIM, GM, MG, MR, CS	No correlation, Strong OME - PE correlation	No HC, Sample features, Few cognitive indices
Tercero et al. (2021)	IA, IB	SCHZ, BD	CR, *ad-hoc* MC indices	low IA, pos. IB, BD > SCHZ	No HC, Sample features, Few cognitive indices, Modified meta-WCST
Jones et al. (2021)	Sadness, IB	SCHZ, BD	CR	SCHZ < BD	No HC, Sample features, Few indices, Modified meta-WCST
Badal et al. (2023b)	IA, Confidence	SCHZ, BD	CR, *ad-hoc* MC indices	BD > SCHZ	No HC, Sample features, Few indices, Modified meta-WCST
Badal et al. (2023a)	IA, IB	HC, SCHZ, BD	CR, *ad-hoc* MC indices	conf-feedb corr. in BD-HC, neg. corr in SCHZ	Sample features, Few indices, Modified meta-WCST
Bampa et al. (2024)	Metac. training	MCI	CR, TE, PE, NPE, PR, CC, TFC, FMS	higher CR, MC indices, lower PE	Sample features, single-blind
Gorora et al. (2024)	IA, IB	SCHZ, BD	CR, CC, TFC, AS, *ad-hoc* MC indices	BD corr. > SCHZ corr.	No HC, Sample features, Few indices, Modified meta-WCST
Fan et al. (2025)	tDCS, rIPFC, IA	SCHZ, SCHZAF	QS, confidence	IA improved, SCHZAF > SCHZ	No HC, Sample features, Few indices, Modified meta-WCST

SCHZ, Schizophrenia; QS, Quantity Score; CC, Completed Categories; PE, Perseverative Errors; PR, Perseverative Responses; TFC, Trials-to-First Category; AS, Accuracy Score; FIM, Free-choice Improvement; GM, Global Monitoring; MG, Monetary Gain; MR, Monitoring Resolution; CS, Control Sensitivity; HC, Healthy Controls; AN, Anorexia Nervosa; TE, Total Errors; APS, Attenuated Psychotic Symptoms; OME, Objective Metacognitive Evaluations; SME, Subjective Metacognitive Evaluations; IA, Introspective Accuracy; IB, Introspective Bias; BD, Bipolar Disorder; CR, Correct Responses; MCI, Mild Cognitive Impairment; NPE, Non-perseverative Errors; FMS, Failures-to-Maintain Set; tDCS, Transcranial Direct Current Stimulation; rIPFC, Right Rostrolateral Prefrontal Cortex; SCHZAF, Schizoaffective Disorder.

Koren et al. (2004) investigate insight in SCHZ patients. The study takes into account several cognitive indices (QS, CC, PE, perseverative responses—PR, and trials-to-first category—TFC) and all metacognitive indices (AS, FIM, GM, MG, MR, CS). They detected a correlation between poor insight and metacognitive deficits, highlighting that metacognitive measures improve insight prediction without affecting cognitive measures. Although this is the study that proposes the meta-WCST, it shows methodological limitations such as the lack of a healthy control group and a small, unbalanced experimental group.

Koren et al. (2005) investigate the “competence to consent” in SCHZ patients, taking into account the same indices as the previous study. In particular, they find a correlation between poor competence to consent and metacognitive deficits. Furthermore, they again observe that metacognitive measures improve the prediction of competence to consent without compromising cognitive measures. Unfortunately, the study shares the methodological limitations of the previous one (no healthy control group, small and unbalanced experimental group).

Koren et al. (2006) integrate the two previous studies by investigating both insight and competence to consent in schizophrenic patients. This study takes into account the same indices as the previous one. Furthermore, it shows the same correlations between insight and consensus competence with metacognitive deficits, confirming that cognitive measures are not compromised. This study shares the same limitations as the previous ones.

Arbel et al. (2013) investigate insight in anorexic patients. This study takes into account only three cognitive indices (QS, CC, PR) and excludes the MG from the six metacognitive indices. They detected that insight impairment is more correlated with metacognitive deficits than with cognitive deficits. Furthermore, the relationship between insight, metacognition, and cognition is mediated by IQ in the meta-WCST. Although these interesting findings, the study shows methodological limitations, such as a clinically heterogeneous and gender-unbalanced sample and the inclusion of few cognitive indices.

Quiles et al. (2014) investigate the impact of metacognitive measures on cognitive measures in a healthy population. They administered a modified version of the WCST (Nelson, 1976) and a modified version of the meta-WCST, thus taking into account only CC and TE and no metacognitive index. Unexpectedly, they detected no significant differences between the basic and metacognitive condition. However, the study considered only two cognitive indices (CC, TE) and ignores other indices that may help to investigate cognitive load and distraction (e.g., NPE).

Koren et al. (2017) investigate the relationship between self-disturbance (SD), cognition, and metacognition in help-seeking adolescents (15.85 ± 0.07 years) with and without attenuated psychotic symptoms. They take in consideration only one cognitive index (CR) and five metacognitive indices (AS, FIM, GM, MR, CS). Unexpectedly, they find a positive correlation between metacognition and SD that is not influenced by APS. However, they hypothesize that the hyper-reflectivity of SD individuals may explain the results. This study lacks a healthy control group and an adequate set of cognitive indices.

Marucci et al. (2019) investigate the relationship between subjective (SME) and objective (OME) metacognitive evaluations in SCHZ patients. Therefore, they administer the meta-WCST to collect OME measures (PE, AS, FIM, GM, MG, MR, CS) and the Subjective Scale to Investigate Cognition in Schizophrenia (SSTICS; Stip et al., 2003) to collect SME measures. Unexpectedly, they detected no correlation between the SSTICS and meta-WCST indices. However, they found a strong negative correlation between PE and the metacognitive indices of the meta-WCST. In addition to common sample limitations (e.g., no healthy control group), the inclusion of only PE severely limits this study.

Tercero et al. 2021) investigate Introspective Accuracy (IA) and Introspective Bias (IB) in SCHZ patients and BD patients. They administer a modified version of the meta-WCST that shows different requests and scores. Therefore, they use only the CR index of the meta-WCST and elaborate *ad-hoc* metacognitive indices. They detect that both groups show impaired IA and positive IB; therefore, they overestimate their performance. However, BD patients perform significantly better than SCHZ patients, probably also because the former show higher feedback sensitivity. On the other hand, the metacognitive indices of SCHZ patients were better correlated with each other than with cognitive performance. Similar to other studies, this one has sample limitations (no healthy control group, unbalanced and heterogeneous experimental groups) and limited data analysis (they only calculated CR as a cognitive index). Finally, they significantly modified the meta-WCST, thus preventing a clear comparison with the original studies.

Jones et al. 2021) investigate the relationship between sadness and IB in SCHZ and BD patients, taking in consideration only CR of the meta-WCST. They found that meta-WCST performance was significantly worse in the never-sad and occasionally-sad SCHZ patients compared to the BD group. This study shares the methodological limitations of others (no control group, unbalanced experimental group, few cognitive and metacognitive indices, a modified meta-WCST that prevents clear comparison with the original studies).

Badal et al. (2023b) investigate the IA in SCHZ and BD patients. They adopt a modified version of the meta-WCST, thus computing the CR and *ad-hoc* metacognitive indices with decomposition analyses. They detect that BD patients modulate their confidence rate based on feedback, while SCHZ patients do so very poorly, and this deficit is distinct from their perseverative behavior. This study shows the same limitations as many others (no healthy control group, clinically heterogeneous and unbalanced samples, and modified meta-WCST poorly comparable with the original studies).

Badal et al. (2023a) investigate the temporal relationships between IA, accuracy judgments, confidence, and feedback processing in SCHZ patients, BD patients, and healthy controls (HC). They adopt a modified version of the meta-WCST, thus calculating CR and *ad-hoc* metacognitive indices with network analyses. They find a stronger correlation between confidence and feedback processing in BD patients and HC compared to SCHZ patients. Moreover, past confidence ratings show a negative correlation with future performance in SCHZ patients. Although this study uses new methods (network analyses), it has the same limitations as the previous one.

Bampa et al. (2024) investigate the efficacy of a Metacognitive Training Program (MTP) in patients with MCI. Therefore, they include the meta-WCST administration into a follow-up design (start, 3 months, 6 months) with a control group (untrained patients) and an experimental group (trained patients). Notably, they take into consideration several cognitive indices (CR, TE, PE, NPE, PR, CC, TFC, FMS), a few metacognitive indices (AS, GM), and *ad-hoc* metacognitive indices. After 3 months, the experimental group showed fewer PR than the control group, while after 6 months, the differential improvement also involved CR and PE. The same trend was observed in metacognitive measures. This is the only study that includes the meta-WCST in a follow-up design, although it has a small sample and a single-blind design.

Gorora et al. (2024) applied new analyses to data collected by (Tercero et al. 2021), thereby measuring how trial accuracy and accuracy judgments predict the trial correctness and accuracy judgment in SCHZ and BD patients. They exploit a time-series analysis including several cognitive indices (CR, CC, TFC), standard metacognitive indices (AS), and *ad-hoc* metacognitive indices. They detected that BD patients show faster improvements in trial accuracy and correct accuracy judgments. In addition, these accuracy judgments strongly influence the subsequent trial correctness and accuracy judgments. In contrast, trial correctness poorly predicts subsequent trial correctness in SCHZ patients, and there is no correlation between previous and subsequent accuracy judgments. Overall, the study suggests that SCHZ patients do not take into consideration external feedback when making decisions. Although the study applies innovative analyses, it inherits both the dataset and the methodological limitations (no healthy control group, clinically heterogeneous samples, few cognitive indices, a modified version of meta-wcst, not easy to compare with the original one).

Fan et al. 2025) investigate the efficacy of brain stimulation (transcranial direct current stimulation; tDCS) applied to a frontal structure (right rostrolateral prefrontal cortex; rIPFC) in order to enhance IA in SCHZ and SCHZAF patients. They only considered the QS index and confidence levels. They observed a performance improvement over time in the experimental group (target stimulation) compared to the control group (no target stimulation). Furthermore, SCHZAF patients performed better than SCHZ patients. Although this is the first study to combine brain stimulation with meta-WCST administration, it has limitations similar to other studies, such as clinically heterogeneous samples, limited cognitive and metacognitive indices, and a version of the meta-WCST that is not fully comparable to the original.

### 3.1 Current limitations for adapting to developmental neuropsychology

The Meta-WCST has the potential to be administered to childhood populations. However, given the high inter-individual and intra-task heterogeneity observed in developmental populations (especially in specific conditions such as ADHD or autism; Capobianco et al., 2025; Jacob et al., 2019), the results (e.g., group-level correlations and index differences; see Table 1) and analyses (e.g., classical aggregated indices; see Section 2.1) derived from adult studies may not be directly suitable for characterizing developmental behaviors. In addition to widespread sampling issues, such as lack of control groups and small sample sizes, we identified three key areas that limit the generalizability of findings to developmental populations and need adaptation: theoretical-computational frameworks, analysis methods, and administration protocols.

First, no study introduces a theoretical-computational framework that describes MC-EF interactions in detail. While (Koren et al. 2006) proposes a theoretical interpretation of task measures and some studies attempt to quantify interactions between factors (e.g., Badal et al., 2023a), none include a neuro-computational framework that accurately interprets interactions between EF latent factors (working-memory decay, decision focus, and so on) and MC latent factors (evaluation history, MC noise, and so on). Furthermore, they do not include a theory-based computational model that produces experimental/clinical predictions and disentangles fine-grained neuropsychological interactions. This limitation negatively impacts the generalizability of previous findings because developmental populations generally show less predictable neuropsychological latent factors and interactions (e.g., motivation and MC; Zheng et al., 2021; Sibley et al., 2019; Bednarz et al., 2020).

Second, except for seminal proposals (Badal et al., 2023a; Gorora et al., 2024), the studies do not delve deeply into the analysis to capture trial-by-trial trends that characterize specific test conditions (e.g., rule changes). Furthermore, most studies consider one or a few cognitive indices, often neglecting some that may reflect key neuropsychological traits (e.g., LTL). We expect that indices commonly used in adult studies would not adequately capture children's higher trial-to-trial variability and less consistent task engagement (e.g., children may show a less consistent approach to confidence estimation than adults; Eberhart et al., 2025).

Finally, since there is no official documentation of the task, the administration procedure is not standardized. Due to the difficulties in conceptualizing specific demands (e.g., confidence assignment) and motivation/attention deficits in typical and atypical populations, we believe that administration/instructions would be unsuitable for children. For example, it is important to explain the metacognitive scoring mechanism (e.g., “counted incorrect sorts” decreases the final score, while “not counted incorrect sorts” do not). Again, the standard feedback stimulus (red or green colored terms “INCORRECT”/“CORRECT”) should be replaced with clearly interpretable emojis.

Overall, these points provide experimental scientists and clinicians with a concise, practical roadmap for developing a developmental version of the Meta-WCST: improved theoretical-computational frameworks, fine-grained trial-by-trial analysis methods, and simplified/accessible administration procedures (e.g., task stimuli and instructions) tailored to children.

## 4 Future directions and applications

We began addressing literature limitations through theoretical, methodological, and experimental approaches. For example, we recently proposed a theoretical framework and a related computational model of the meta-WCST, thereby validating it with healthy and clinical populations (Granato et al., 2025b). In our study, we introduced a modeling approach called the ‘neuropsychological digital-twin method', which supports the creation of digital twins for neuropsychological profiling and intervention predictions. On the other hand, we are developing automated neuropsychological pipelines (task administration, scoring, modeling), whose theoretical-computational proposals are validated with several human populations in healthy, neurodivergent, and clinical conditions (Granato et al., 2020; Granato and Baldassarre, 2021; Granato et al., 2022; Granato and Baldassarre, 2024; Granato et al., 2025a,b). Notably, these pipelines contain trial-by-trial analyses and are also being integrated with EBRAINS (Granato, 2025), a widely distributed platform that fosters integration within the European research ecosystem (Schirner et al., 2022; Appukuttan et al., 2023). Finally, we are developing and validating a “developmental meta-WCST” to conduct experimental investigations with children showing typical and atypical development. We aim to administer this task to four groups of children (control, ADHD, SLD, and mixed profiles) to generate normative and clinical data. Specifically, we will extract several new indices (e.g., reaction times for each trial) to identify suitable markers that may distinguish clinical from typical developmental conditions. These data should provide experimental validation for the task and serve as an effective tool for researchers and clinicians.

### 4.1 Application contexts

A developmental Meta-WCST can be applied across several scientific, educational, and clinical fields. For example, it can support the investigation of debated topics such as the interacting maturation of EF and MC in neurodevelopmental populations (Roebers, 2017; Dörrenbächer-Ulrich et al., 2024). On the other hand, it can support comparisons between different ages within the same neurodivergent population. Educational contexts would benefit from this tool. For example, a number of studies highlight that learning programs should take into account MC and EF features (Orsolini et al., 2022; West Gaskins and Pressley, 2007; Rivella et al., 2024; Meltzer et al., 2021). Additionally, parents could benefit from orientation programs informed by this tool (Capobianco and Costa, 2024). Finally, this test would serve as a key tool for neuropsychological assessments in developmental clinical contexts. In particular, it would support key early neuropsychological screenings targeting EF-MC (Orsolini et al., 2023), providing psychotherapists with key information to plan individualized and effective interventions (Capobianco and Costa, 2024; Capobianco and Cerniglia, 2018), thereby improving the quality of life in adulthood (Campbell and Burchinal, 2007).
